# Severe Clade I (subclade Ib) mpox in advanced HIV complicated by multidrug-resistant bacterial superinfection and ocular involvement: a case report from Uganda

**DOI:** 10.1186/s41182-026-01010-9

**Published:** 2026-06-24

**Authors:** Raymond Ernest Kaweesa, Brenda Nairuba, Geoffrey Odoch, Rodney Abraham Tumusiime, Deborah Mukisa, Annie Daphine Ntabadde, Violet Ankunda, Pontiano Kaleebu, Christopher Nsereko, Jennifer Serwanga

**Affiliations:** 1 Viral Pathogens Theme, Immunology, MRC/UVRI & LSHTM Uganda Research Unit, Plot 51-59 Nakiwogo Road, Entebbe, Uganda; 2 Uganda Virus Research Institute (UVRI), 51-59 Nakiwogo Road, P.O. Box 49, Entebbe, Uganda; 3 London School of Hygiene & Tropical Medicine, Keppel Street, London, WC1E 7HT UK; 4 Entebbe Regional Referral Hospital, Kampala Road, P.O. Box 29, Entebbe, Uganda

**Keywords:** Mpox, HIV, Advanced immunosuppression, Resource-limited settings, Opportunistic infections, Uganda, Antibiotic resistance, Ocular management

## Abstract

**Background:**

Mpox in people living with HIV (PLHIV) with advanced immunosuppression is poorly understood in sub-Saharan Africa, where high HIV prevalence intersects with limited access to mpox-specific antivirals. Urgent evidence is needed to guide clinical management and improve outcomes for this vulnerable population in resource-limited settings with outbreaks.

**Case presentation:**

We report a 30-year-old Ugandan man with advanced HIV (CD4 55 cells/µL, HIV-1 RNA 645,000 copies/mL) who presented with 2 weeks of widespread mucocutaneous disease, circumferential necrotic penile ulceration with severe dysuria, and periocular involvement. Mpox was confirmed by real-time PCR from multiple anatomical sites, with sequencing identifying Clade I (subclade Ib) infection. Longitudinal sampling showed consistently low *Ct* values in skin and genital lesions despite rising salivary *Ct* values, indicating differential compartmental viral clearance. Serial pus cultures from ulcerated lesions yielded multi-resistant gram-negative bacilli and coagulase-negative staphylococci, with sustained resistance to first-line agents throughout the clinical course. Meropenem remained the only antimicrobial to which isolates were consistently susceptible across all sampling points, suggestive of evolving polymicrobial wound colonisation under antibiotic selection pressure.

**Management and outcome:**

Empirical ceftriaxone and metronidazole proved ineffective. Culture-guided escalation to intravenous meropenem, along with intensive wound care, pain management, ocular prophylaxis, and resumption of antiretroviral therapy (ART), resulted in gradual clinical improvement. The patient was discharged after 4 weeks with healed skin lesions and mild residual ocular discomfort. At 3 months, immune reconstitution was observed (CD4 173 cells/µL; HIV-1 RNA 72 copies/mL) with complete functional recovery.

**Conclusions:**

Severe Clade Ib (subclade Ib) mpox in advanced HIV can present with genital necrosis, ocular involvement, persistent lesion-site viral burden, and multidrug-resistant bacterial superinfection. Even in the absence of specific antivirals, favourable outcomes can be achieved through integrated HIV care, early culture-guided antimicrobial stewardship, and proactive ocular management.

## Introduction

Mpox, caused by the mpox virus (MPXV), has re-emerged as a global health threat, with Clade I (subclade Ib) now driving sustained human-to-human transmission across Africa [1–8]. Recent outbreaks have demonstrated a disproportionate burden of severe disease in people living with HIV (PLHIV), particularly those with advanced HIV disease, defined by a CD4 cell count less than 200 cells/µL or WHO stage 3 or 4 in adults and adolescents [9]. Reported complications in this population include necrotic genital ulcers, bacterial sepsis, sight-threatening ocular issues, and death [10–15]. Despite the expanding overlap between Clade Ib mpox and advanced HIV, detailed case-based evidence from sub-Saharan Africa remains limited, particularly in settings, where viral suppression is often suboptimal, and access to antiviral therapies and specialised care is limited [16–18].

Importantly, severe mpox in PLHIV in Africa unfolds against a backdrop of rising antimicrobial resistance (AMR) in common skin and soft-tissue pathogens, as well as constrained access to broad-spectrum agents, which complicates the management of secondary bacterial infections [18–20]. However, detailed case-based descriptions linking host immune status, lesion viral dynamics, bacterial co-infection, AMR patterns, and real-world management decisions are rare. What remains poorly defined is how severe Clade Ib mpox evolves in PLHIV with advanced immunosuppression in African hospitals, particularly when complicated by antimicrobial resistance and limited access to antivirals and specialist care.

We report a severe, prolonged case of Clade Ib mpox in a 30-year-old Ugandan man with advanced HIV (CD4 55 cells/µL), complicated by genital necrosis, ocular involvement, and multidrug-resistant superinfection, who ultimately recovered after multidisciplinary care and immune reconstitution. This case underscores key considerations for diagnosis, antimicrobial stewardship, ART optimization, and prevention of ocular sequelae in resource-limited settings. It emphasises the need to integrate mpox preparedness within HIV programs and investigates how severe mpox presents and progresses in PLHIV, as well as what management strategies are effective in such contexts.

## Patient information

A 30-year-old Ugandan man with a known history of HIV infection presented with a 2-week history of progressive mucocutaneous lesions and systemic symptoms. The illness began with intermittent fevers and chills, followed by the appearance of pustular lesions on the face that spread rapidly to the scalp, trunk, limbs, and genital region. He developed marked genital pain with a circumferential necrotic ulcer of the penile shaft and associated dysuria. Ocular involvement then emerged with bilateral conjunctival injection, photophobia, and excessive tearing.

The patient lived in a stable monogamous heterosexual relationship and worked as a taxi driver. No known contact with a confirmed mpox case was reported, although direct contact was presumed as the likely mode of transmission. He reported longstanding non-adherence to ART (tenofovir, lamivudine, and dolutegravir).

He was managed at Entebbe Regional Referral Hospital Isolation Unit, a specialised referral facility for epidemic-prone infectious diseases in Uganda. The facility is equipped with dedicated isolation infrastructure, multidisciplinary infectious disease expertise, microbiological support, and established outbreak response capabilities, distinguishing it from most healthcare facilities in the country.

The patient provided written informed consent for the publication of this case report, including permission for the anonymisation of clinical data and de-identified photographic documentation.

## Clinical findings

**Fig. 1:**
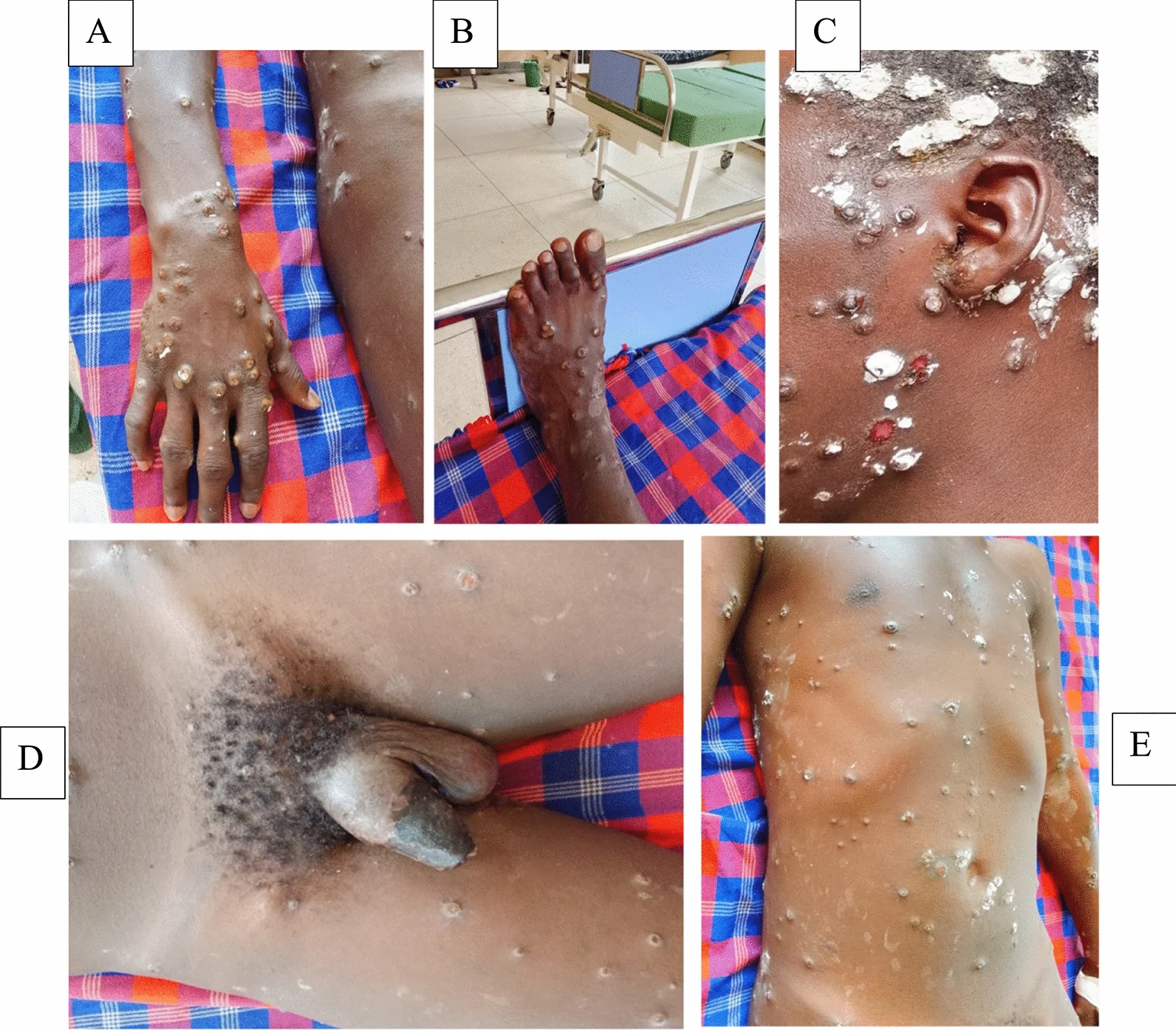
Clinical manifestations of severe mpox in a patient with advanced HIV-1. Sequential images showing dermatological and mucocutaneous manifestations of severe mpox in a 30-year-old man with advanced HIV (CD4 55 cells/μL). Early lesions on the hand and arm display classic umbilicated pustules and crusted nodules (**A**). Involvement of the lower limb includes papulopustular and crusted eruptions (**B**). Facial and scalp lesions are accompanied by peri-auricular secondary bacterial infection (**C**). Extensive anogenital disease features a necrotic penile ulcer, bilateral inguinal lymphadenopathy, and disseminated lesions over the groin and limbs (**D**). Widespread trunk eruptions at different stages of development highlight the severity and systemic spread of cutaneous disease (**E**)

The patient was febrile (38.2 °C) with widespread umbilicated pustules and crusted lesions involving the face, scalp, neck, trunk, extremities, and genitalia. A circumferential necrotic ulcer encircled the penile shaft, accompanied by bilateral tender inguinal lymphadenopathy (Fig. 1). Ocular examination revealed bilateral conjunctival injection, photophobia, and excessive tearing. The timeline of illness and key clinical events is summarised in Table 1.

**Table 1:** Timeline of illness and management

Illness day	Event
Days 0–10	Initial symptoms begin with fever, chills and facial pustules. Lesions start spreading to the scalp, trunk, limbs, and genital area Progressive worsening of mucocutaneous disease. Severe genital pain develops. A circumferential necrotic penile ulcer forms. Eye symptoms emerge with redness, tearing, and photophobia
Days 11–12	Lesions become purulent. Deterioration leads to admission. Extensive pustular and crusted lesions noted. Mpox strongly suspected
Day 12	Mpox confirmed by PCR from skin, genital, and saliva swabs. First *Ct* values recorded. First bacterial pus swab collected. Empiric ceftriaxone and metronidazole and supportive care started
Days 13–14	Persistent severe genital and cutaneous disease. Ocular discomfort worsens
Day 15	No improvement on empiric antibiotics. Second pus swab collected
Day 21	Bacterial culture shows multidrug-resistant organisms only susceptible to meropenem Antibiotics escalated to meropenem. Due to periocular involvement, acyclovir eye ointment and ciprofloxacin eye drops added to prevent complications. ART restarted with adherence support
Days 24–28	Gradual clinical improvement. Lesions re-epithelialise
Day 29	Discharged in stable condition after 4 weeks of inpatient care
Month 3 follow-up	Full recovery. Lesions healed with scarring only. No active ocular disease. CD4 rises to 173 cells/µL. HIV viral load suppressed. Returned to work and baseline functioning

## Diagnostic assessment

Mpox was suspected upon presentation due to widespread umbilicated pustules, a circumferential necrotic penile ulcer, tender lymphadenopathy, and ocular irritation. The main clinically excluded differentials included herpes simplex virus infection, chickenpox, syphilitic genital ulcer disease, chancroid, and severe bacterial cellulitis. However, the lesion pattern showing widespread umbilicated pustules at various stages of development, the extent of mucocutaneous involvement, prominent lymphadenopathy, severe genital ulcer, and ocular irritation were most consistent with mpox. Herpes simplex and chancroid were considered less likely given the disseminated pustular form and systemic distribution. In contrast, varicella-zoster virus typically does not cause severe genital necrosis or marked lymphadenopathy in adults. Syphilis was considered, but it is unlikely to account for the acute necrotic lesions and widespread skin involvement.

PCR testing from skin, genital, and saliva swabs confirmed MPXV infection. Laboratory evaluations revealed profound immunosuppression, with a CD4 T-cell count of 55 cells/µL and an HIV plasma viral load of 645,000 copies/mL. Hematologic results showed mild lymphopenia (1.0 × 10^3^/µL; reference range 1.2–3.7 × 10^3^/µL) with preserved total leukocyte counts. Platelet count, white cell differentials, and other complete blood count parameters were within normal ranges.

Serum chemistry revealed hyponatremia (131 mmol/L; reference range 136–145 mmol/L) and hypochloremia (90.7 mmol/L; reference range 98–107 mmol/L), with normal renal and liver function test results. Notably, C-reactive protein was markedly elevated at 95.7 mg/L (reference range 0.00–5 mg/L), consistent with systemic inflammation. Urinalysis revealed trace hematuria (1 +) and mild proteinuria (1+).

Assessment of secondary bacterial infection incorporated both clinical and laboratory findings. Features supporting localised bacterial superinfection included progressive purulent discharge, worsening necrotic ulceration, and severe localised pain. In contrast, there was no evidence of severe systemic infection: the patient remained haemodynamically stable without hypotension or persistent tachycardia beyond that attributable to fever, and there was no clinical or biochemical evidence of end-organ dysfunction or metastatic foci of infection suggestive of haematogenous dissemination. The total leukocyte count remained within the normal range, although a blunted inflammatory response cannot be excluded in the context of advanced HIV-associated immunosuppression (CD4 55 cells/µL). Systemic inflammation was nonetheless evident, with a markedly elevated C-reactive protein concentration (95.7 mg/L). However, blood cultures were not performed as part of routine clinical management, limiting definitive exclusion of bacteraemia. Overall, the combined clinical and laboratory profile was most consistent with localised soft-tissue bacterial superinfection of necrotic mpox lesions without clear evidence of bloodstream infection or sepsis.

Microbiological assessment was undertaken to further characterise the suspected secondary bacterial infection. Bacterial isolates from purulent mpox lesions were identified using standard aerobic culture, Gram staining, and biochemical methods supplemented by automated identification. Antimicrobial susceptibility testing was performed by EUCAST-standardised disk diffusion, with results interpreted using current EUCAST clinical breakpoints. Coagulase-negative staphylococci were considered clinically significant when repeatedly isolated from purulent lesions in the presence of systemic inflammatory features.Following laboratory confirmation of mpox, the patient received counselling on transmission risk, infection prevention measures, and partner notification as part of routine clinical care. Through patient interview, his regular sexual partner was identified and notified by the clinical team, and counselled regarding symptom recognition and indications for medical review. She remained asymptomatic throughout the reported follow-up period.

Formal contact tracing beyond the identified close contact was not undertaken as part of this case investigation, which focused primarily on clinical management during the outbreak response. The patient’s occupation as a taxi driver involved frequent transient public interactions, making identification and follow-up of potential occupational contacts impractical. Consequently, broader community contact investigations were not feasible within available outbreak-response resources.

## Virological characterisation and clade assignment

MPXV DNA was detected from multiple sites (skin, genital, and saliva swabs) using a real-time PCR assay targeting the F3L gene. The F3L gene was selected as a diagnostic target, because it is highly conserved within MPXV and is widely used in validated molecular assays for sensitive and specific clinical detection [19]. For clade determination, high-quality lesion DNA (CT ≤ 15) underwent amplicon-based whole-genome sequencing on an Illumina platform, followed by consensus generation using a validated in-house pipeline. Clade assignment using Nextclade and phylogenetic placement against a curated global reference dataset confirmed infection with Clade I, subclade Ib, without evidence of unusual divergence. This finding situates the case within the ongoing Clade Ib epidemic in East and Central Africa and supports comparison with a recent series of severe mpox cases in advanced HIV.

## Mpox viral load dynamics and antimicrobial resistance trends

**Fig. 2:**
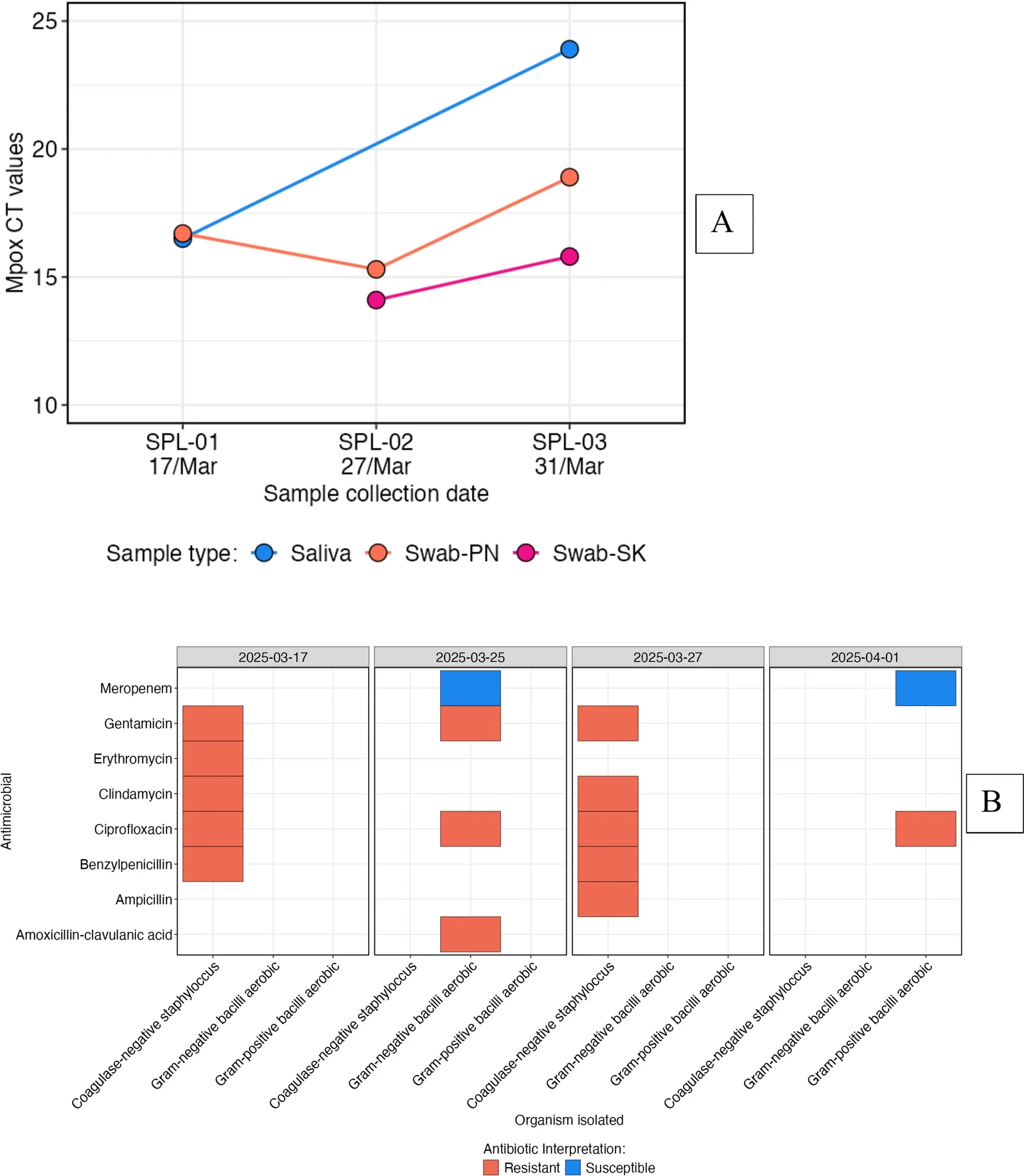
Longitudinal dynamics of mpox viral load and antimicrobial susceptibility patterns in the patient. **A** Line plot showing MPXV PCR cycle threshold (*Ct*) values over three sampling timepoints (days 12, 22, and 26 post-infection). Data are stratified by specimen type: saliva (blue), penile swab (orange), and skin swab (pink). Each point represents the *Ct* value for a given specimen type at each timepoint, connected by lines to visualise temporal trends. **B** Illustrates a stacked bar graph of antibiotic susceptibility results from serial bacterial cultures isolated from an mpox patient over four timepoints (March 17 to April 1, 2025). Each bar represents the dominant isolate per timepoint. The chart presents susceptibility interpretations (red = resistant, blue = susceptible) of isolates, *coagulase-negative Staphylococcus*, gram-negative bacilli, and gram-positive bacilli, against eight antibiotics across multiple classes. Resistance was most pronounced early (March 17), with broad non-susceptibility across β-lactams, aminoglycosides, macrolides, and fluoroquinolones. Emergent susceptibility to meropenem and ciprofloxacin was observed from March 25 onward, indicating dynamic shifts in pathogen composition or treatment response

Mpox viral load was monitored over three timepoints, day 12, day 22, and day 26 post-infection, corresponding to March 17, March 27, and March 31, respectively (Fig. 2A). Viral dynamics were monitored with real-time PCR using saliva, penile (Swab-PN), and skin (Swab-SK) specimens (Fig. 2A). *Ct* values from saliva samples increased progressively from day 12 to day 26, indicating a declining systemic viral burden. In contrast, penile (Swab-PN) and skin (Swab-SK) samples consistently exhibited lower *Ct* values, reflecting persistently higher local viral loads at mucocutaneous sites. While persistent low *Ct* values in lesions suggest delayed local viral clearance, the relationship between *Ct* values and infectivity in mpox remains incompletely defined.

Antimicrobial susceptibility testing of bacterial isolates from serial pus swabs collected on days 12, 20, 22, and 27 post-infection revealed extensive multidrug resistance (Fig. 2B). Serial swab cultures were obtained in response to persistent purulent discharge and limited clinical response to empiric therapy. Culture results revealed the isolation of diverse bacterial species on different days, including gram-negative bacilli and coagulase-negative staphylococci (Fig. 2B). Notably, the patient exhibited multidrug resistance across most agents tested, with Meropenem as the sole consistently susceptible antimicrobial. The evolving microbial profile and resistance patterns underscore the dual clinical challenge of sustained viral replication in cutaneous niches, alongside the evolving bacterial resistance that occurs during mpox infection.

## Therapeutic interventions

The patient was started on empiric intravenous ceftriaxone and metronidazole for presumed secondary bacterial infection, which lasted 10 days, along with intravenous fluids and antipyretics for symptom relief. Since culture results revealed multidrug-resistant gram-negative organisms and coagulase-negative staphylococci, which were susceptible to meropenem, antimicrobial therapy was escalated to intravenous meropenem at 500 mg every 8 h for 5 days. Local wound care involved debridement and daily sterile dressings. Due to periocular involvement, acyclovir eye ointment and ciprofloxacin eye drops were given to prevent keratitis and additional ocular complications. After screening for opportunistic infections, ART was resumed with counseling and close inpatient adherence support. Daily clinical reviews directed management of pain, hydration, nutrition, and infection control measures.

## Outcome

The patient gradually improved after switching to meropenem and restarting ART. Fever resolved, pain decreased, and the mucocutaneous lesions started to dry and re-epithelialise. The necrotic penile ulcer healed without new breakdowns, and no additional systemic issues arose. He completed 4 weeks of inpatient care and was discharged in stable condition. At the 3-month review, he achieved full functional recovery.

All skin lesions healed with residual scarring, and the penile ulcer was fully closed. He reported mild ocular discomfort, but ophthalmology assessment showed no active disease or vision loss. His HIV markers showed good response, with CD4 count rising from 55 to 173 cells/µL and viral load dropping from 645,000 to 72 copies/mL. He had returned to work and maintained regular clinic visits with improved ART adherence. Ongoing follow-up will focus on monitoring immune recovery, ocular health, and the risk of late complications.

## Discussion

This case illustrates a fulminant and multisystem presentation of Clade Ib mpox in a 30-year-old man with advanced HIV, characterised by disseminated mucocutaneous disease, circumferential penile necrosis, periocular involvement, multidrug-resistant bacterial superinfection, and prolonged lesion viral burden. In the absence of mpox-specific antivirals, recovery was achieved through coordinated multidisciplinary care combining culture-guided antimicrobial therapy escalation, structured wound and ocular management, and ART reinitiation with adherence support, culminating in immune reconstitution at 3 months.

A central observation is the interaction between profound immunosuppression and delayed, compartmental viral clearance. Despite rising *Ct* values in saliva over time, persistently low *Ct* values in penile and skin lesions suggested sustained local MPXV DNA burden at lesion-rich anatomical sites, consistent with impaired viral control in advanced HIV and prior reports linking low CD4 counts to severe and prolonged mpox disease courses [4, 13, 20, 21]. Clinically, this pattern has practical implications: systemic improvement or reduced saliva viral load may not reliably indicate lesion clearance or reduced infectivity risk in advanced HIV, and it reinforces the need for repeated lesion-based assessment, prolonged infection prevention and control counselling, and site-appropriate sampling to monitor clinical trajectory.

Secondary bacterial infection emerged as a major contributor to morbidity within a broader AMR context. Serial pus swab cultures from ulcerated lesions yielded gram-negative bacilli and coagulase-negative staphylococci resistant to multiple first-line agents, with preserved susceptibility to meropenem and intermittently ciprofloxacin. This mirrors wider AMR trends in African hospitals [22–24] and underscores that empirical ceftriaxone-based regimens may provide inadequate coverage in severe mpox with secondary bacterial infection. Accordingly, early culture and susceptibility testing should inform targeted therapy, with carbapenems reserved for culture-confirmed, life-threatening infections and de-escalated promptly when narrower options are available.

However, an important interpretive caveat is that cultures from open cutaneous and genital mpox lesions are prone to contamination by resident and transient microbiota. Consequently, recovery of these organisms, such as coagulase-negative staphylococci, cannot, in isolation, distinguish invasive infection from surface colonisation, and should be interpreted within a broad clinical context, particularly in anatomically colonised mucocutaneous sites, where polymicrobial flora are expected.

Diagnostic accuracy for secondary bacterial infection may be improved through deep tissue biopsy or sterile pus aspiration sampling rather than superficial lesion swabs. Where clinically feasible, tissue biopsies better reflect the organisms present within infected tissue, while pus aspirates provide a less invasive alternative for accessible collections. In contrast, cultures from superficial swabs are more susceptible to contamination by colonising organisms and may not reliably distinguish surface colonisation from invasive infection [25]. These limitations have important implications for antimicrobial stewardship, as culture results from open wounds should be interpreted alongside systemic clinical features, including local inflammatory signs, systemic inflammatory markers, haemodynamic status, and response to therapy, rather than microbiological results in isolation.

Ocular involvement represents another clinically important but operationally challenging complication of mpox, particularly in resource-limited settings with constrained ophthalmology access [17, 18, 26]. In this patient, early periocular symptoms prompted topical antiviral and antibacterial prophylaxis alongside ophthalmology referral. While residual discomfort persisted, no visual loss or active ocular disease was documented at follow-up. The clinical message is not that topical prophylaxis is universally required, but rather that routine ocular screening should be embedded into mpox assessment, with a low threshold for early referral when keratitis cannot be excluded, and pragmatic interim measures when specialist review is delayed. In resource-limited settings, preventing avoidable ocular sequelae may depend more on standardised screening pathways and early escalation than on access to advanced therapeutics.

This case further highlights the clinical opportunity created by rapid re-engagement with HIV care. ART re-initiation with adherence support was temporally associated with sustained improvement and immune recovery (CD4 55 to 173 cells/µL; HIV-1 RNA 645,000 to 72 copies/mL). Although immune reconstitution inflammatory syndrome (IRIS) remains a relevant concern when restarting ART in advanced immunosuppression, delayed ART optimisation likely perpetuates vulnerability to prolonged mpox disease, bacterial superinfection, and related complications. Accordingly, severe mpox should be conceptualised and managed as an integrated HIV-associated complication requiring coordinated virological, microbiological, ophthalmological, and public health management. Clinical guidance should, therefore, incorporate HIV staging, ART optimisation, antimicrobial stewardship frameworks, structured wound and ocular assessment pathways, and referral mechanisms for severe disease. At a policy level, mpox preparedness should be integrated within existing HIV and epidemic preparedness systems rather than implemented through parallel vertical programmes.

Beyond individual clinical management, this case also underscores persistent inequities in access to mpox diagnostics, therapeutics, and vaccines in endemic African regions, which remain a critical barrier to outbreak control and equitable global health security. Strengthening preparedness will require expanded access to validated molecular diagnostics, antivirals, such as tecovirimat, and vaccines for high-risk populations and exposed contacts. Additional priorities include strengthening laboratory and surveillance systems, expanding clinical trial inclusion for African populations, and establishing equitable procurement and distribution mechanisms for emerging interventions. Sustainable international partnerships and integration within existing epidemic-response infrastructure will be essential to reduce morbidity and mortality during future outbreaks.

Finally, the confirmation of Clade I, subclade Ib, situates this case within the ongoing East and Central African epidemic and aligns with reports of severe disease and high mortality among PLHIV in this context [3, 12]. In the absence of mpox-specific antivirals, this case supports a pragmatic, replicable care pathway for severe mpox in advanced HIV: prompt molecular confirmation from lesion sites; early HIV staging with CD4 count, HIV viral load, and opportunistic infection screening; routine pus culture and susceptibility testing for necrotic lesions; structured wound and ocular assessment with early referral pathways; timely ART optimisation with adherence support and monitoring for IRIS; and antimicrobial stewardship guided by susceptibility data.

This report  is limited by it's  single-patient design, , the absence of blood cultures to exclude occult bacteremia, lack of tissue biopsies or histopathology, and limited long-term follow-up. Nonetheless, it provides context-specific evidence from a regional referral hospital in Uganda and highlights modifiable factors that may improve outcomes in PLHIV with severe mpox.

In conclusion, severe Clade Ib mpox in advanced HIV can manifest with extensive genital necrosis, ocular involvement, persistent lesion viral burden, and multidrug-resistant bacterial superinfection. However, this case demonstrates that favorable outcomes are achievable even without specific antivirals when mpox is managed as an integrated HIV complication, combining early diagnosis, culture-guided antimicrobial therapy, meticulous wound and ocular care, and rapid ART optimisation. Embedding these elements within African HIV programmes and hospital care pathways is essential to reduce mpox-related morbidity and mortality among immunosuppressed populations at the intersection of overlapping epidemics.

## Patient perspective

“The illness was very painful and frightening, especially the severe sores on my genitals and the fear that I might lose my eyesight. I was scared and uncomfortable for many days. I am grateful for the close medical care I received, the regular checks, and the encouragement that helped me restart my HIV treatment. As my wounds slowly healed and my pain reduced, I felt great relief. This experience has taught me how important it is to take my HIV medicine every day to avoid becoming this sick again.”

## Learning points

- Advanced HIV disease is a critical host factor determining mpox severity; CD4 count at diagnosis should be mandatory in all mpox assessments, as counts below 200 cells/µL, and particularly below 100 cells/µL, consistently predict protracted, necrotic, and potentially fatal disease
- Secondary bacterial infection is an underappreciated complication of severe mpox in sub-Saharan Africa. Pus culture with susceptibility testing at admission, interpreted alongside systemic inflammatory markers, should guide early targeted escalation and inform empirical antibiotic choices in settings with high endemic AMR burden.
- Structured ophthalmological assessment should be embedded in mpox admission protocols. Periocular involvement carries the risk of corneal ulceration and permanent visual loss that is preventable with early recognition and appropriate ocular therapy.
- Clade Ib mpox is clinically distinct from the 2022 global Clade IIb outbreak and requires Africa-specific protocols, regional guideline development, and equitable access to diagnostics, antivirals, and vaccines.

## Data Availability

The datasets generated during the current study are not publicly available due to patient confidentiality considerations but are available from the corresponding author on reasonable request, subject to institutional and ethical approvals.
